# Can you hear us now? The impact of health-care utilization by rare disease patients in the United States

**DOI:** 10.1038/s41436-021-01241-7

**Published:** 2021-06-28

**Authors:** Angela A. Navarrete-Opazo, Maharaj Singh, Ainslie Tisdale, Christine M. Cutillo, Sheldon R. Garrison

**Affiliations:** 1 Advocate Aurora Health, Advocate Aurora Research Institute, Milwaukee, WI USA; 2 School of Dentistry, Marquette University, Milwaukee, WI USA; 3 National Center for Advancing Translational Sciences, National Institutes of Health, Bethesda, MD USA

## Abstract

**Purpose:**

The vast majority of rare diseases (RDs) are complex, disabling, and life-threatening conditions with a genetic origin. RD patients face significant health challenges and limited treatments, yet the extent of their impact within health care is not well known. One direct method to gauge the disease burden of RDs is their overall cost and utilization within health-care systems.

**Methods:**

The 2016 Healthcare Cost and Utilization Project (HCUP) databases were used to extract health-care utilization data using International Classification of Diseases, Tenth Revision (ICD-10) codes.

**Results:**

Of 35.6 million national hospital weighted discharges in the HCUP Nationwide Inpatient Sample, 32% corresponded to RD-associated ICD-10 codes. Total charges were nearly equal between RDs ($768 billion) compared to common conditions (CCs) ($880 billion) (*p* < 0.0001). These charges were a result of higher charges per discharge and longer length of stay (LOS) for RD patients compared to those with CCs (*p* < 0.0001). Health-care cost and utilization was similarly higher for RDs with pediatric inpatient stays, readmissions, and emergency visits.

**Conclusion:**

Pediatric and adult discharges with RDs show substantially higher health-care utilization compared to discharges with CCs diagnoses, accounting for nearly half of the US national bill.

## INTRODUCTION

Rare diseases (RDs) are a global health-care problem with an estimated 400 to 700 million people affected worldwide [1–3]. Currently, the number of RDs has been suggested to be more than 10,000 [4]; these diseases are often serious, quality of life-limiting, and potentially life-threatening. Most RDs have some level of genetic involvement, with 72–80% of these conditions having an identified gene or genes [5, 6]. In the United States, RDs are defined as any condition affecting fewer than 200,000 individuals, which collectively affects an estimated 33 million people [7]. In Europe, the European Medicines Agency (EMA) specifies a prevalence of less than 5 in 10,000 people (~75 million), and in Japan the Ministry of Health, Labour and Welfare defines RDs as any condition affecting less than 50,000 individuals in the country (~12.5 million) [8].

Patients living with RDs experience significant health, psychosocial, occupational, and financial burden. The financial burden of RDs includes both direct (medical and nonmedical) and indirect costs. Direct medical or health-care cost burden can reach millions of dollars annually for certain rare diseases, with cost drivers that include hospitalizations and emergency visits, medications, dental health, palliative care, outpatient visits, insurance cost and reimbursement, rehabilitation care, home health care, assistive devices, social services, and caregivers [9–15]. RD patients typically experience significant diagnostic delay averaging over 5 years [16, 17], and requires the involvement of a knowledgeable and comprehensive clinical care team to determine a definitive diagnosis.

The overall health-care utilization by pediatric and adult populations with RDs in the United States has not been well documented. Evidence is emerging that RDs may have a disproportionate and substantial impact within health care that well exceeds RD patient prevalence. A recent study analyzed health-care utilization of pediatric patients with 919 genetic diseases and found a marked increase in those patients with one or more genetic diseases [18]. Aggregate total charges for suspected genetic diseases, many of which are rare, in 2012 accounted for ~$57 billion (46%) of the “national bill” for pediatric patients [18]. However, pediatric patient inpatient stays account for only a small component of the total impact of rare disease in health care, and a current and broad inquiry of RDs is necessary.

The present study is a comprehensive investigation of health-care utilization of adult and pediatric patients with RDs compared to those without a RD in the United States. These data span all inpatient, readmission, and emergency department data within the same year (2016) using the Healthcare Cost and Utilization Project (HCUP) database. Here we report the widespread economic impact of RDs in the United States across all demographics. This amplifies the need to incorporate cost-saving measures and improved health-care access for those affected by rare disease.

## MATERIALS AND METHODS

### Data source

The 2016 Nationwide Inpatient Sample (NIS), Kids’ Inpatient Database (KID), Nationwide Readmissions Database (NRD), and Nationwide Emergency Department Sample (NEDS) HCUP data were used to extract health-care utilization and cost data for 1,645 International Classification of Diseases, Tenth Revision (ICD-10) codes linked to RDs. The ICD-10 code list linked to 1,645 RDs, or features of them, was primarily provided by Orphanet [19], a worldwide database dedicated to providing information on rare diseases and orphan drugs [20], and does not include ICD-10 codes for all rare diseases. Common conditions (CCs) were defined as any condition not included in the RD ICD-10 list. Of the 1,645 ICD-10 codes and linked RDs, 1,091 have some level of genetic involvement (66.3%) as determined using OMIM and Orphanet [19, 21]. The 2016 HCUP database was selected because, at the time of analysis, it is the most recent set of data that includes the KID database, which is released every 4 years.

The NIS database is the largest publicly available all-payer inpatient health-care database designed to produce US national estimates of inpatient utilization, access, charges, and outcomes. For the year 2016, NIS contains an administrative and demographic data sample that includes an estimated 97% of discharges in the United States from hospitals in 46 states and the District of Columbia [22], from which a random 20% sample is derived. An estimated 35.6 million hospital weighted discharges were identified in the 2016 NIS HCUP database, of which 11 million correspond to the 1,645 ICD-10 codes from suspected RD diagnoses of record (32%) and 24 million correspond to CC discharges (31%) (Table 1). The types and locations of hospitals from which the NIS data were derived are described in Table S1.

**Table 1:** Health-care utilization and demographic characteristics, National Inpatient Stay (NIS).

	Rare disease %	Common condition %	*P* value
***n***	11,289,703	24,378,608	
**Age (years)**	58.3	44.7	<0.0001
**Neonate at admission**	0.7	0.9	<0.0001
**Sex**			<0.0001
Female	51.3	59.2	
Male	48.7	51.3	
**Race and ethnicity**			<0.0001
White	68.2	64.1	
Black	15.7	15.0	
Hispanic	9.8	13.4	
Asian or Pacific Islander	2.8	3.2	
Native American	0.6	0.7	
Other	3.0	3.6	
**Payer**			<0.0001
Private	23.8	33.0	
Medicare	53.7	33.1	
Medicaid	16.9	26.0	
Self-pay	2.8	4.4	
No charge	0.3	0.3	
Other	2.6	3.2	
**Location**			<0.0001
Large metro (central)	29.6	30.3	
Large metro (fringe)	24.8	23.6	
Medium metro	20.8	20.7	
Small metro	9.3	9.2	
Micropolitan	9.0	9.2	
Nonmetro, nonmicropolitan	6.6	7.0	
**Income quartile by ZIP code ($)**			<0.0001
1–42,999	29.9	31.1	
43,000–53,999	25.2	25.5	
54,000–70,999	24.1	23.8	
71,000+	20.8	19.6	
**Discharge disposition**			<0.0001
Routine	56.4	75.0	
Transfer to short-term hospital	2.7	1.6	
Transfer to other facility	20.0	11.1	
Home health care	16.0	10.0	
Left against medical advice	1.1	1.3	
Died	3.9	1.0	
**Elective**			<0.0001
Elective admission	17.6	23.2	
Nonelective admission	82.4	76.8	
**Procedures per discharge (number)**			<0.0001
0	38.2	38.8	
1–5	52.7	57.7	
6–10	7.3	3.1	
11–15	1.8	0.4	
**Transfer in**			<0.0001
Not transferred in/newborn	90.2	93.5	
From acute care hospital	7.1	4.7	
From another type of health facility	2.8	1.8	
**Transfer out**			<0.0001
Not a transfer	77.3	87.3	
To acute care hospital	2.7	1.6	
To another type of health facility	20.0	11.1	
**Hospital division**			<0.0001
New England	5.1	4.4	
Middle Atlantic	14.0	13.8	
East North Central	15.9	15.0	
West North Central	6.8	7.0	
South Atlantic	21.1	20.4	
East South Central	6.6	6.9	
West South Central	10.7	12.4	
Mountain	6.1	6.3	
Pacific	13.5	13.8	
Missing values not displayed			

Demographic characteristics, NIS, weighted estimate.

The HCUP-KID database contains an all-payer, national sample of pediatric inpatient discharges for patients younger than 21 years of age [23]. The KID database includes conditions and treatments that are normally difficult to treat, including rare disease and uncommon treatments (e.g., organ transplants), allowing for health-care utilization to be thoroughly investigated [24]. In the year 2016, a total of 6 million weighted discharges were identified for children less than 21 years of age in the 2016 KID HCUP database, of which 1 million (21%) correspond to RDs versus 5 million to CCs (79%).

The HCUP NRD is drawn from 27 HCUP State Inpatient Databases (SID) and can be used to create estimates of 30-day, all-cause hospital readmissions rates and their associated costs [25]. Overall, there were 16 million readmissions in the year 2016, of which 5 million correspond to patients with RDs (32%) and 11 million (68%) to CCs patients.

The NEDS database is drawn from the SID and State Emergency Department Databases (SEDD) [26]. For the year 2016, 36 states and the District of Columbia contributed to the database, representing 78% of all US emergency department visits. In 2016, there were approximately 178 million ED visits in the United States, of which 14 million (10%) correspond to patients with RDs and 130 million (90%) to patients with CCs. All *n*’s in each database are estimates and analysis of each data element may vary due to missing values.

### Data elements

Clinical and nonclinical hospitalization data elements were extracted from the HCUP NIS, KID, NRD and NEDS databases for selected ICD-10 codes linked to RDs diagnosis. Sample nonclinical data elements included (1) demographic information (sex, age, race, median household income, patient location), (2) primary payer, (3) hospital characteristics (location and region), and (4) total charges. Sample clinical related information included (1) primary diagnosis, (2) discharge status, (3) origin and disposition of the patient, (4) type of admission, (5) hospital discharges, and (6) length of stay (LOS). The following additional data elements were extracted from the KID database: (1) neonatal age and (2) uncomplicated vs. complicated in-hospital birth; from the NRD database: (1) transfer to rehabilitation, evaluation, or other aftercare; and from the NEDS database: (1) total number of ED visits. The number of discharges was provided from total discharges and the *n* was not further provided for each data element, which varied for each data element due to missing content. The description of each HCUP data element is included in table S2.

### Data analysis

A retrospective analysis of 2016 HCUP NIS, KID, NEDS, and NRD was conducted. The estimated prevalence data was adjusted for the US population using the discharge weight variable (DISCWT) to minimize the margin of error and to reflect all 50 states across the United States. To establish the impact of rare disease, patients in the RD cohort were included if they had a suspected rare disease diagnosis from the list of 1,645 ICD-10 codes within the first 15 diagnoses. Descriptive statistics of the weighted national estimates were used to summarize the results. Continuous variables are presented as means and standard error of the mean (SEM). Categorical variables were shown in percentage values. Two-sided tests (chi-square and Fisher’s exact for categorical measures, and Student’s *t* tests for continuous measures) were used for RD and CC comparisons. Data presented in Tables 1–4 used separate chi-square tests and there were no multiple comparisons. All statistical tests used in this paper were two-tailed. Statistical significance was considered for *p* < 0.05. All analyses and appropriate weighted estimates were calculated using SAS (version 9.4; SAS Institute Inc., Cary, NC).

**Table 2:** Health-care utilization and demographic characteristics, Kids Inpatient Database (KID).

	Rare disease %	Common condition %	*P* value
***n***	1,200,587	5,065,496	
**Age (years)**	4.7	3.9	<0.0001
**Sex**			<0.0001
Female	46.8	52.6	
Male	53.2	47.4	
**Race**			<0.0001
White	47.3	51.6	
Black	19.9	15.4	
Hispanic	20.7	21.3	
Asian or Pacific Islander	5.1	4.9	
Native American	0.8	0.9	
Other	6.1	6.1	
**Payer**			0.0003
Private	41.1	43.5	
Medicare	0.5	0.4	
Medicaid	51.1	48.7	
Self-pay	3.0	4.3	
No charge	0.1	0.1	
**Location**			0.0001
Large metro (central)	34.6	32.7	
Large metro (fringe)	24.3	23.4	
Medium metro	20.5	20.7	
Small metro	8.1	8.7	
Micropolitan	7.5	8.6	
Nonmetro, nonmicropolitan	5.0	5.8	
**Elective**			<0.0001
Elective admission	15.1	7.0	
Nonelective admission	84.9	93.0	
**Discharge disposition**			<0.0001
Routine	88.1	96.4	
Transfer to short-term hospital	4.0	1.1	
Transfer to other facility	1.7	0.8	
Home health care	4.7	1.4	
Left against medical advice	0.2	0.2	
Died	1.3	0.1	
**Mortality (within group)**			<0.0001
Neonate (overall)	0.8	0.6	
<1 year	1.7	0.40	
1–4 years	0.9	<0.1	
5–9 years	0.8	<0.1	
10–13 years	0.9	<0.1	
14–16 years	1.2	<0.1	
**Emergency services**			<0.0001
No ED services of record	75.6	82.2	
Record of ED services	24.4	17.8	
**Transfer in**			<0.0001
Not transferred in/newborn	89.6	95.4	
From acute care hospital	9.1	3.8	
From another type of health facility	1.3	0.8	
**Transfer out**			<0.0001
Not a transfer	94.3	98.2	
To acute care hospital	4.0	1.1	
To another type of health facility	1.6	0.8	
**In-hospital birth**			<0.0001
Transferred in from acute/other	58.2	35.5	
Born inside same hospital	41.8	64.5	
**In-hospital birth**			<0.0001
Complicated	94.4	54.6	
Uncomplicated	5.6	45.4	
**Income quartile by ZIP code ($)**			<0.0001
1–42,999	30.5	30.6	
43,000–53,999	24.9	24.2	
54,000–70,999	24.1	24.0	
71,000+	20.6	19.1	
**Hospital region**			0.9618
Northeast	17.0	16.4	
Midwest	21.6	21.6	
South	38.8	39.2	
West	22.7	22.9	
Missing values not displayed			

Demographic characteristics, KID, weighted estimate.

*ED* emergency department.

**Table 3:** Health-care utilization and demographic characteristics, Nationwide Readmissions Database (NRD).

	Rare disease %	Common condition %	*P* value
*n*	5,155,566	11,053,961	
Age (years)	57.8	44.0	<0.0001
Sex			<0.0001
Female	51.4	59.2	
Male	48.6	40.8	
Payer			<0.0001
Private	23.7	33.9	
Medicare	54.2	25.2	
Medicaid	16.5	33.1	
Self-pay	2.4	3.9	
No charge	0.4	0.5	
Location			<0.0001
Large metro (central)	26.6	26.8	
Large metro (fringe)	27.3	26.1	
Medium metro	20.1	20.2	
Small metro	9.8	10.4	
Micropolitan	9.2	9.4	
Nonmetro, nonmicropolitan	7.1	7.2	
Elective			<0.0001
Elective admission	17.0	21.7	
Nonelective admission	83.0	78.3	
Discharge disposition			<0.0001
Routine	59.0	77.2	
Transfer to short-term hospital	1.2	0.6	
Transfer to other facility	17.8	9.7	
Home health care	17.0	10.2	
Left against medical advice	1.0	1.3	
Died	4.0	1.0	
Income quartile by ZIP code ($)			<0.0001
1–42,999	29.1	30.4	
43,000–53,999	25.6	25.4	
54,000–70,999	25.6	25.2	
71,000+	19.7	18.9	
Diagnoses per discharge (number)			<0.0001
0	0.0	0.1	
1–5	9.2	38.2	
6–10	22.1	29.8	
11–15	27.3	17.4	
Procedures per discharge (number)			<0.0001
0	29.1	30.4	
1–5	25.6	25.4	
6–10	25.6	25.2	
11–15	19.7	18.9	
Same day event			<0.0001
No transfer	95.9	98.0	
Two or more different hospitals	3.8	1.9	
Hospital state residency			<0.0001
Resident	94.3	95.7	
Nonresident	5.7	4.3	
Rehabilitation transfer			<0.0001
No transfer	99.7	99.8	
To rehabilitation, evaluation or other	0.3	0.2	
Missing values not displayed			

Demographic characteristics, NRD, weighted estimate.

**Table 4:** Health- care utilization and demographic characteristics, National Emergency Department Sample (NEDS).

	Rare disease %	Common condition %	*P* value
*n*	14,072,499	130,770,243	
Age (years)	58.3	37.7	<0.0001
Sex			<0.0001
Female	53.4	55.7	
Male	46.6	44.3	
Died during ED visit			<0.0001
Did not die	97.4	99.6	
Died in ED	0.2	0.1	
Died in hospital	2.3	0.1	
Payer			<0.0001
Private	22.4	29.2	
Medicare	52.1	19.9	
Medicaid	18.1	33.8	
Self-pay	4.4	12.1	
No charge	0.3	0.4	
Location			<0.0001
Large metro (central)	28.5	30.2	
Large metro (fringe)	23.4	20.3	
Medium metro	21.6	20.8	
Small metro	9.9	10.5	
Micropolitan	9.7	10.5	
Nonmetro, nonmicropolitan	6.4	7.2	
Outcome of ED visit			<0.0001
Treated and released	46.3	89.1	
Admitted to same hospital	51.1	9.0	
Transferred to short-term hospital	2.3	1.6	
Destination unknown	0.2	0.2	
Discharge disposition			<0.0001
Routine	43.6	86.3	
Transfer to short-term hospital	2.3	1.6	
Transfer to other facility	1.5	1.2	
Home health care	0.5	0.2	
Left against medical advice	0.7	1.5	
Admitted as inpatient at ED hospital	51.1	9.0	
Income quartile by ZIP code ($)			<0.0001
1–42,999	30.8	35.2	
43,000–53,999	25.6	26.9	
54,000–70,999	22.2	20.4	
71,000+	19.6	15.8	
Diagnoses per discharge (number)			<0.0001
0	0.0	0.0	
1–5	23.3	80.3	
6–10	28.5	13.4	
11–15	21.7	3.8	
Missing values not displayed			

Demographic characteristics, NEDS, weighted estimate.

*ED* emergency department.

The study followed the required research practices based on the Agency for Healthcare Research and Quality (AHRQ)’s recommendations including (1) identifying observations as hospitalization events rather than unique patients, (2) not performing state-level or physician-level analyses, (3) not using nonspecific secondary diagnosis codes to infer in-hospital events, (4) using survey-specific analysis methods allowing for weighting of estimates to generate national estimates with an accompanying measure of variance of the estimate, and (5) not including data from any condition with ten or fewer encounters.

## RESULTS

To better understand the economic impact and health-care utilization of RD, a comprehensive analysis of inpatient, readmission, and emergency visits in 2016 was conducted. A RD in a patient’s record was the single biggest predictor of health-care services, and included duration, type, and cost of that utilization when comparing to CCs. These factors were analyzed in detail to provide a comprehensive assessment of the impact of RD in health care. The individual conditions included in the study, as well as average age, LOS, and total charges are included in Table S3.

### Health-care utilization

**Fig. 1:**
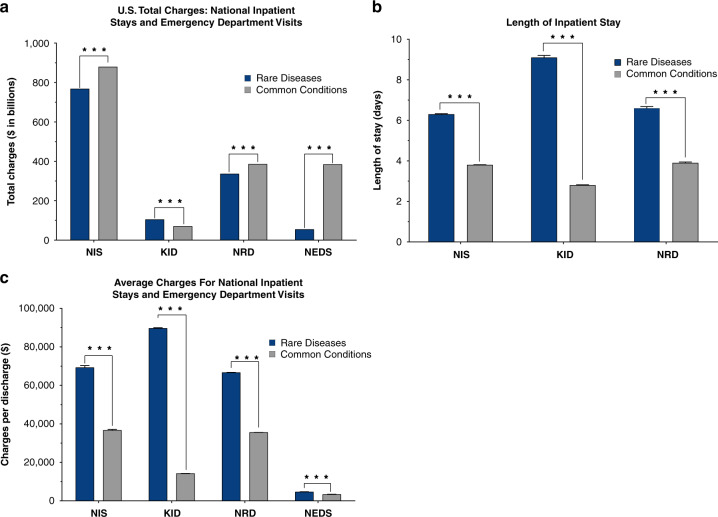
Estimated total charges for US health-care utilization of rare diseases (RDs) compared to common conditions (CCs). RD patient cost burden was significantly higher than patients with common conditions (CCs) across all databases except Nationwide Emergency Department Sample (NEDS). (**a**) Estimated total charges for the Nationwide Inpatient Sample (NIS) were $768 billion (**b**) for RDs and $880 billion for CCs. Estimated total charges for RDs for Kids’ Inpatient Database (KID), Nationwide Readmissions Database (NRD), and NEDS were $105 billion, $337 billion, and $55 billion, respectively. Estimated total charges for CCs for KID, NRD, and NEDS were $70 billion, $385 billion, and $384 billion, respectively. All comparisons were *p* < 0.0001 and estimated total chargers are shown. (**b**) Estimated charges per discharge for RDs was higher than for CCs. Estimated average charge per discharge for NIS was $69,275 ± 1004 compared to $36,718 ± 389 for CCs (*p* < 0.0001). Estimated average charge per discharge for KID was $89,681 ± 289 for RDs compared to $14,226 ± 23 for CCs (*p* < 0.0001). Estimated average charge per discharge for NRD was $66,675 ± 98 for RDs compared to $35,585 ± 28 for CCs (*p* < 0.0001). Estimated average charge per discharge for NEDS was $4,670 ± 108 for RDs compared to $3,397 ± 76 for CCs (*p* < 0.0001). Mean cost per discharge shown with error bars indicating standard error. (**c**) Estimated inpatient length of stay (LOS) for RDs was longer in each Healthcare Cost and Utilization Project (HCUP) database evaluated (NIS, KID, NRD, NEDS) when compared to CCs. Estimated LOS for NIS was 6.3 days compared to 3.8 days for CCs (*p* < 0.0001). Estimated average charge per discharge for KID was 9.1 days compared to 2.8 days for CCs (*p* < 0.0001). Estimated average charge per discharge for NRD was 6.6 days compared to 3.9 days for CCs (*p* < 0.0001). Mean LOS is shown with error bars indicating standard error.

Health-care utilization was captured using total aggregate inpatient charges, LOS, and charges per inpatient visit. Hospital inpatient visits reflect the single largest cost source in health care, and therefore, provide the most significant component of RD health-care utilization. Overall, total aggregate charges were $768 billion for RDs and $880 billion CCs in the 2016 NIS HCUP database, a remarkable difference of nearly $111 billion (Fig. 1a; *p* < 0.0001) despite RD patients accounting for a very small percentage of the overall US population [1–3].

The present study shows significant differences in health-care utilization between RD and CC discharges. RDs had a longer LOS (6.3 days) compared to CCs (3.8 days) (Fig. 1b; *p* < 0.0001), and nearly double the average total charges per discharge ($69,275 ± 1004) compared to CCs ($36,718 ± 389) (Fig. 1c; *p* < 0.0001).

Pediatrics are of great interest for rare disease. Despite representing only 20.9% of total inpatient stays for children less than 21 years of age, total aggregate charges were approximately $34 billion higher for RDs ($105 billion) than CCs ($70 billion) (Fig. 1a; *p* < 0.0001). The mean total charge per patient was $89,618 ± 289 for RDs and $14,226 ± 23 for CCs (Fig. 1c, *p* < 0.0001). The mean LOS was over three times longer for RDs (9.1 days) compared to CCs (2.8 days) (Fig. 1b; *p* < 0.0001).

RDs also had a disproportionate impact on readmission and emergency visits. Data from the NRD showed that patients with RDs had a lower readmission rate (32%) but higher total charges per readmission than patients with CCs ($66,675 ± 98 vs. $35,585 ± 28, *p* < 0.0001) (Fig. 1c). The impact of RDs was much lower in the emergency department (ED), accounting for 9.7% of overall ED visits. The total charges per visit were greater for RDs ($4,670 ± 108) than CCs ($3,397 ± 76, *p* < 0.0001) (Fig. 1c), resulting in $55 billion for RDs compared to $384 billion for CCs (Fig. 1a; *p* < 0.0001).

### Clinical data

In addition to total charges and LOS, the overall impact of RD is more fully understood by the number of procedures per inpatient stay, patients’ transfer among facilities, and mortality rate. In the NIS database, RD patients have more inpatient procedures, with 9.1% having 6–15 procedures compared to 3.5% for CCs. Furthermore, fewer RD patients were routinely discharged (e.g., home) (56.4%) compared to CC patients (75.0%). Instead, RD patients were frequently transferred to another facility (22.7%) or required home health care (16.0%). Unfortunately, four times as many RD patients died (3.9%) during their inpatient stay compared to CC (1.0%) patients (*p* < 0.0001) (Table 1).

In the pediatric population, RD patients had fewer routine discharges (88.1%) compared to CC patients (96.4%) (*p* < 0.0001), and more RD patients were transferred to another facility or home health care (5.7%) compared to CC discharges (1.9%). Mortality rates were 13 times higher in RDs (1.3%) compared with CCs (0.1%) (*p* < 0.0001; Table 2), with mortality remaining significantly higher across all age groups. One of the most striking differences between RD and CC was specific to a subset of patients at birth. When determining those patients who were born within the admitting hospital to those newborns who were transferred from another acute care hospital or health-care facility, RD patients were found to have taken a very different pathway. Approximately 58% of RD patients were transferred from another hospital or care facility compared to only 36% of CC patients. Of those born in the same hospital, 94% of RD births were complicated (e.g., cesarean section, birth trauma) compared to 55% of CC patients. Upon further evaluation of this striking difference of birth complications revealed racial disparity. Normal births were similar in breakdown by race between RD and CC patients.

For ED visits, only 56.3% of RD patients were treated and released, and 51.1% were admitted to the same hospital. In contrast, most of the CC visits were treated and released (89.1%) with only 9.0% admitted to the hospital (*p* < 0.0001). Moreover, 12 times more RD patients (2.3%) died in the ED or later as an inpatient in the hospital, compared with those with CCs (0.2%) (Table 4).

### Demographics

Given the large number of discharges, it was important to better understand the patient population within the sample. Overall, the frequency of RDs in males was 7.9% higher than in CCs in the NIS database. Surprisingly, the average age was higher with RD, 58.3 years, compared to 44.7 years for CCs. There was also a difference in race, with White patients more frequently diagnosed with a RD (68.2%) than CC (64.1%), and fewer Hispanic RD patients (9.8%) compared to CC (13.4%). Private insurers were the primary payer for CCs (33.0%) while public payers, Medicare and Medicaid, were the primary payer for RD visits (70.6%) (Table 1).

Children admitted with a RD were on average 4.7 years old, whereas children with a CC averaged 3.9 years old (*p* < 0.0001). Most patients were White for both RD (47.3%) and CCs (51.6%). For both RDs and CC, the primary payer was Medicaid. However, when RDs were further evaluated by race, a much higher percentage of White RD patients used private insurance (54.4%) compared to Black (22.0%) and Hispanic (22.8%) patients (Table S4). The NIS, KID, NRD, and NEDS demographic data are further described in Tables 1–4.

## DISCUSSION

There is a clear and immediate public health interest relating to the socioeconomic impact and management of RDs to develop sustainable health policy measures. Systematic quantification of the economic burden of RDs at the national level will help illuminate the direct financial consequences of rare conditions in the health system. We captured various types of health-care utilization HCUP data, the largest all-payer databases of discharges in the United States, to estimate the economic burden of patients suffering from RDs by analyzing the inpatient, readmission, and ED cost burden within health care. Overall, discharges with RD-associated codes show disproportionately higher health-care cost and utilization across all age groups compared with discharges with CC diagnoses.

### Rare diseases have a massive impact in US health care

People with RDs disproportionately utilized health-care systems, particularly with inpatient stays where RD patients had more discharges and readmissions, longer LOS, and greater charges per inpatient stay. Here, we report that for the year 2016, overall national total charges were similar for RDs compared to all other CCs. Moreover, pediatric charges were $34 billion greater for RDs than CCs. Limited reports of the disproportionate cost burden of RD have emerged in recent years. In Hong Kong, inpatient health care of 467 RDs was shown to account for 4.3%, or HKD 1,594,339,530 ($204,402,504), of overall inpatient costs in 2015–2016 [14]. Likewise, a systematic literature review of the cost of illness studies assessed the indirect and direct cost of ten rare conditions in the European Union in the year 2010 [15]. Annual direct cost for patients with RDs ranged from €3,858 ($4,334) for scleroderma to €23,066 ($25,911) for hystiocytosis [15]. While these studies analyze far fewer conditions compared to the current study with limited scope, as well as report on non-US health-care systems, the health-care utilization and financial impact is still large and suggests issues that expand well beyond the United States.

The cost of RD highlights significant concern around payer utilization. With the average cost per patient and total cost for RDs being incredibly high, payers and best practices for delivering quality health care may influence patient outcomes and long-term national spending. In the current study we report that overall, RDs accounted for an estimated $768 billion in inpatient costs alone in 2016, with each inpatient stay averaging $69,275. Following the initial inpatient stay, these costs may increase significantly, with increased rates of readmission and transfer to expensive care options (e.g., discharge to home health care or another facility). As a result, the demands placed on RD patients and their families to manage the cost burden are daunting, particularly when 70.6% require a public payer (Medicare or Medicaid). Moreover, private insurers lack meaningful and universal strategies to reduce RD cost burden, even at the level of orphan drugs that are prescribed to RD patients [27, 28]. In sum, there remains a significant opportunity to streamline RD clinical management and broaden effective treatments to reduce cost burden and improve patient outcomes.

### How can the economic impact and system utilization of RDs be reduced through improved management?

Costs associated with a rare disease include frequent and multidisciplinary care expenses, costly procedures, and expensive medications [17, 29]. Diagnostic delays of RDs contribute to this financial burden [17]. The majority of RD patients must leave their health-care system (e.g., out-of-network, out-of-geographical region) and visit at least 3–10 doctors prior over the course of years before receiving a definitive diagnosis and beginning treatment [17, 30]. These diagnostic inefficiencies not only result in the potential of leading to costly and unnecessary treatments [31, 32], but they also can push the patient beyond treatment windows, a major concern for life-shortening conditions. For example, delayed diagnosis has been reported to result in poorer outcomes and substantially increased cost for the rare conditions Krabbe disease and severe combined immunodeficiency [9, 33, 34]. This may also contribute to the high average cost ($89,539) and duration (9.1 days) of pediatric RD inpatient stays reported in the current study. Therefore, to provide the most effective therapies for RD patients, multiple approaches should be taken to address the issues of diagnostic delay and initiating best-in-class treatments for RD patients, such as improved clinician education, continued development of new drugs and gene therapies, and reduced time to diagnosis due to expanded newborn screening.

### Study limitations

The overall cost of RDs is likely larger than the current HCUP utilization analysis. HCUP estimate charges likely underestimate the true costs of these conditions because they do not factor direct costs such as professional (e.g., physician, dentist, and other clinicians) fees, indirect costs (e.g., lost work productivity), and secondary downstream health-care effects.

HCUP databases are useful for giving estimates on a national scale. There are, however, several limitations of HCUP sample data: (1) the frequencies represent hospital discharges, and not patients, and thus, recurrent hospitalizations by the same patient appear as distinct observations; (2) the prevalence data may be affected by hospital coding; (3) databases do not capture outpatient encounters, and the full health-care utilization of patients suffering from RDs is underrepresented; (4) data do not represent the complete universe of all discharges in the United States since not all states participate; (5) hospital charges represent the bill that is sent to the payer, not the actual cost to the hospital which may vary, depending on reimbursement, if any; (6) the number of rare conditions included in this study primarily correspond to ICD-10 codes provided by Orphanet, which is far from the nearly 7,000–10,000 rare conditions currently described; (7) patients with undiagnosed rare conditions are not included in the RD cohort; (8) conditions are heterogeneous and genetic basis may affect only a disease subpopulation; (9) the ICD-10 codes used also include CC, which are unable to be separated from RDs, and thus are included in the health-care utilization and cost data reported here; and (10) we did not distinguish conditions in Table S3 that are indicated as having a genetic basis due to genetic susceptibility, genetic role in the phenotype, or disease-causing somatic mutation(s) from those with disease-causing germline mutation(s).

## Conclusions

The cost of RDs needs to be calculated to better allocate resources and to find ways to ameliorate individual and societal costs. Resources should be allocated not according to the prevalence of a certain disease, but rather according to where intervention yields the most cost-efficient value. This study demonstrates that during the year 2016, the total national cost of RDs was disproportionate and considerably greater than CCs. Pediatric and adult populations with RDs had longer hospitalizations, more charges per admission, more readmissions, and more mortality than CC patients. Improvements in patient management and health-care utilization strategy may lead to substantial improvement to clinical care and decreased cost burden. Thus, expanded newborn screening tests, health-care-focused artificial intelligence, and other approaches to detect these conditions early in the disease course must be developed and incorporated into the clinical decision-making process to streamline patient care and reduce cost.

## Supplementary information

Supplementary table legend

Supplementary table 1

Supplementary table 2

## Data Availability

Data sets are available upon request to the corresponding author (S.R.G.) and can be shared after consulting our local institutional review board and the Agency for Healthcare Research and Quality.

## Code availability

Computer code used to generate data be accessed from the corresponding author.
