# Supplementary material for: Can you hear us now? The impact of health-care utilization by rare disease patients in the United States
Source: Genet Med. 2021 Jun 28;23(11):2194–201. doi: 10.1038/s41436-021-01241-7 (PMC8553605; doi:10.1038/s41436-021-01241-7)
Supplement: Supplementary file 1 — Supplementary table legend [file 41436_2021_1241_MOESM1_ESM.pdf]

Supplementary Table Legend

Table S1: Description and location of hospitals from National Inpatient Stay (NIS) database.

Table S2: NIS, KID, NRD and NEDS HCUP data elements definitions.

Table S3: List of the 2,118 ICD-10 codes used for data analysis. Average age, average length of stay and average total charges per visit, count and reported genetic involvement are included for each condition. This is an unweighted sample, and conditions with under 11 discharges are not included per HCUP guidelines.

Table S4: NIS and KID payer stratified by race.

Supplementary Table 1. Hospital of health care utilization	
**Category**	**%**
NIS	
**Hospital Region**	
Northeast	13.0
Midwest	30.1
South	38.1
West	18.8
**Teaching Hospital**	
Rural	37.2
Urban Non-Teaching	31.7
Urban Teaching	31.1
**Hospital Bedsize**	
Small	50.5
Medium	20.0
Large	24.5

Supplementary Table 4. Payer stratified by race and ethnicity

NIS	White		Black		Hispanic		Asian or Pacific Islander		Native American		Other	
**Payer**	**RD (%)**	**CC (%)**	**RD (%)**	**CC (%)**	**RD (%)**	**CC (%)**	**RD (%)**	**CC (%)**	**RD (%)**	**CC (%)**	**RD (%)**	**CC (%)**
Private	24.2	35.3	19.0	23.1	20.7	25.0	32.8	47.5	17.1	21.3	28.7	19.2
Medicare	60.2	39.9	45.2	28.2	35.9	17.9	41.4	18.5	40.0	23.7	37.7	35.8
Medicaid	10.8	17.9	28.6	39.0	34.4	47.1	20.2	26.0	35.0	44.5	25.6	35.4
Self-pay	0.0	0.4	0.0	0.1	0.1	0.1	0.0	0.1	0.0	0.0	0.0	0.1
**KID**	**White**		**Black**		**Hispanic**		**Asian or Pacific Islander**		**Native American**		**Other**	
**Payer**	**RD (%)**	**CC (%)**	**RD (%)**	**CC (%)**	**RD (%)**	**CC (%)**	**RD (%)**	**CC (%)**	**RD (%)**	**CC (%)**	**RD (%)**	**CC (%)**
Private	54.4	54.8	22.0	23.2	22.8	24.5	57.6	57.7	21.7	21.8	39.7	40.6
Medicare	0.5	0.4	0.8	0.5	0.3	0.2	0.4	0.3	0.5	0.2	0.3	0.3
Medicaid	38.8	37.5	70.6	69.0	68.0	68.1	33.0	33.0	72.2	69.4	51.6	50.9
Self-pay	0.0	0.5	0.0	0.0	0.0	0.0	0.1	0.1	0.0	0.0	0.0	0.0
